# Immune function (serum IL-4 and IL-5), nutritional status, and clinical outcomes in children with bronchial asthma after vitamin D supplementation

**DOI:** 10.5937/jomb0-56915

**Published:** 2025-08-21

**Authors:** Xiangli Xiao, Ranran Wang, Li Qin

**Affiliations:** 1 Wuhan Red Cross Hospital, Department of Pediatric, Wuhan, Hubei, China

**Keywords:** IL-4 and IL-5, bronchial asthma, pediatric massage, vitamin D, immune function, nutritional status, prognosis, inflammatory cytokines, IL-4 i IL-5, bronhijalna astma, pedijatrijska masaža, vitamin D, imunska funkcija, nutritivni status, prognoza, inflamatorni citokini

## Abstract

**Background:**

This study explores the combined therapeutic effects of pediatric massage and vitamin D supplementation on the nutritional status, immune function (serum IL-4 and IL-5), and clinical outcomes in children with Bronchial asthma (BA).

**Methods:**

A total of 60 children diagnosed with BA were randomly assigned to one of two groups: a control group receiving conventional treatment alone and an experimental group receiving conventional treatment alongside pediatric massage and daily vitamin D supplementation. Both groups were monitored over two months for changes in clinical symptoms, immune markers (CD 3+, CD4+, CD4+/CD8 + ratio), nutritional protein levels (albumin, transferrin, prealbumin, and total protein), and recurrence rates.

**Results:**

The experimental group showed significantly faster symptom relief and improved immune function than the control group. This included enhanced immune markers, such as increased CD3+ and CD4+ counts and an improved CD 4+/CD 8+ ratio. Nutritional status also improved, as evidenced by higher levels of albumin, transferrin, prealbumin, and total protein. Additionally, the recurrence rate in the experimental group was notably lower (10%) compared to the control group. The experimental group also exhibited reduced levels of inflammatory cytokines, including IL-4 and IL-5, suggesting a beneficial effect on immune modulation.

**Conclusions:**

The combination of pediatric acupressure and vitamin D supplementation can improve immune function and inflammatory response in children with BA, which is of clinical value.

## Abstract (sr)

**Uvod:**

Ova studija ispituje kombinovane terapeutske efekte pedijatrijske masaže i suplementacije vitaminom D na nutritivni status, imunsku funkciju (serumski IL-4 i IL-5) i kliničke ishode kod dece sa bronhijalnom astmom (BA).

**Metode:**

Ukupno 60 dece sa dijagnozom BA je nasumično raspoređeno u jednu od dve grupe: kontrolnu grupu, koja je primala samo konvencionalni tretman, i eksperimentalnu grupu, koja je uz konvencionalni tretman dobijala i pedijatrijsku masažu i dnevnu suplementaciju vitaminom D. Obe grupe su praćene tokom dvomesečnog perioda radi procene promena u kliničkim simptomima, imunološkim markerima (CD 3+, CD 4+, odnos CD 4+/CD 8+), nivoima nutritivnih proteina (albumin, transferin, prealbumin i ukupni proteini) i stopi recidiva.

**Rezultati:**

Eksperimentalna grupa pokazala je značajno brže ublažavanje simptoma i poboljšanu imunsku funkciju u poređenju sa kontrolnom grupom. To je uključivalo poboljšane imunološke markere, poput povećanja broja CD3+ i CD4+ ćelija i poboljšanog odnosa CD4+/CD 8+. Takođe je zabeleženo poboljšanje nutritivnog statusa, što se ogledalo u višim nivoima albumina, transferina, prealbumina i ukupnih proteina. Pored toga, stopa recidiva u eksperimentalnoj grupi bila je značajno niža (10%) u poređenju sa kontrolnom grupom. Eksperimentalna grupa je takođe imala smanjene nivoe inflamatornih citokina, uključujući IL-4 i IL-5, što ukazuje na povoljan efekat na imunsku modulaciju.

**Zaključak:**

Kombinacija pedijatrijske akupresure i suplementacije vitaminom D može poboljšati imunsku funkciju i inflamatorni odgovor kod dece sa BA, što ima klinički značaj.

## Introduction

Bronchial asthma (BA) is a common airway disease worldwide, characterised by chronic airway inflammation and recurrent episodes [1]. According to a worldwide epidemiological survey, the average incidence of BA is about 1.24%, showing an increasing trend in recent years [2]. The global mortality of BA is about 0.19-0.44/1O0,000, which has decreased compared to 2006. However, the potential threat of recurrent episodes still deserves clinical attention [3]. BA is more common in children under 14 years of age, with malnutrition-induced immune and metabolic dysfunction believed to be the major trigger. The mortality rate is generally higher in children due to their underdeveloped tracheal function and increased vulnerability to inflammatory processes [4].

The pathogenesis of BA is heavily influenced by the dysregulation of the immune system, particularly through the activation of various inflammatory cytokines, such as interleukins (ILs). IL-4, IL-5, IL-10, and IL-13 are key cytokines involved in the immune response, playing a significant role in the recruitment and activation of eosinophils, a hallmark of the allergic inflammatory response seen in asthma [5] [6]. Elevated levels of IL-4 and IL-13, for example, promote IgE production and the activation of mast cells, leading to airway hyperresponsiveness and remodelling. IL-5, on the other hand, is involved in eosinophil recruitment and activation, further contributing to airway inflammation [7]. IL-10, on the other hand, is an important anti-inflammatory factor that can inhibit the production of pro-inflammatory cytokines and maintain immune homeostasis, thereby alleviating the progression of BA [8]. Clinical studies have demonstrated that IL-10 local replacement therapy significantly improves BA progression [9]. Given these roles, targeting these interleukins and their pathways represents a promising therapeutic strategy for asthma.

At present, there is no complete cure for BA in clinical practice. Treatment strategies focus on controlling inflammation and improving airway function. Clinically, it is required not only to ameliorate pathological changes in children but also to pay attention to their nutritional status, which can impact immune function and inflammatory responses, thus reducing the possibility of recurrence [10]. Vitamin D is an essential nutrient with significant immunomodulatory effects, and it has been shown to influence the expression of several interleukins. Vitamin D can modulate the Th1/Th2 balance by suppressing the production of IL-4, IL-5, and IL-13 while promoting the release of anti-inflammatory cytokines like IL-10, thereby reducing airway inflammation [11]. Research has also shown that vitamin D supplementation can help balance immune responses, maintain calcium and phosphorus homeostasis, and support the health of the respiratory system by reducing inflammation [12].

Moreover, Chinese pediatric massage offers a non-invasive approach that can further enhance the immune function in children. Pediatric massage techniques, which include gentle manipulation and the stimulation of acupuncture points, have been shown to improve qi and blood circulation, relieve asthma symptoms, and enhance the body's inflammatory responses [13]. This makes massage an excellent complementary therapy, particularly in children with BA who may benefit from nutritional and immune system support.

Reports on pediatric nudging combined with vitamin D in BA are rare. Therefore, the current study will provide new references and guidelines for the treatment strategies of BA by focusing on the effects of pediatric nudging combined with vitamin D supplementation on clinical symptoms and inflammatory markers of the immune system, including interleukins, in children with BA.

## Materials and methods

### Study population

First, we used the PASS software to calculate the minimum sample size in the case of = 0.05, which showed that a minimum of 30 study participants were needed in each group. This study selected 60 children with BA who were treated to our hospital from November 2020 to July 2022. It randomised them to a control group (n = 30) and an experimental group (n = 30) for conventional treatment and conventional treatment plus vitamin D therapy, respectively. The random assignment method was the lottery grouping method, and all study participants were unaware of their grouping. The study was conducted in strict accordance with the *Declaration of Helsinki* after obtaining the approval of the Ethics Committee of our hospital, and all guardians of the study subjects signed an informed consent form.

### Eligibility and exclusion criteria

Inclusion criteria: Patients who met the diagnostic guidelines for BA [14], with stable vital signs, aged between 4-12, and no other serious respiratory diseases were included. Exclusion criteria: Patients with congenital dysplasia or chronic diseases other than BA (such as kidney, liver, endocrine, metabolic or nervous system diseases), as well as those who were taking vitamin D supplements, antiepileptic drugs, or systemic steroid diseases that might affect vitamin D levels, were excluded.

### Treatment methods

Both groups were given anti-infection, phlegmresolving treatment, and oxygen inhalation. In addition, they were treated with oral montelukast sodium tablets (CSPC Pharmaceutical Group Co., Ltd., H20203046), 5-10 mg/time/day before bedtime. Moreover, massage was performed by rehabilitation physicians as follows: opening Tianmen, pushing Kangong, pushing Taiyang, pressing Zongjin, and forked pushing Hand-Yinyang 24-30 times each; tonifying the kidney meridian for 400 times, tonifying the lung meridian for 350 times, tonifying the spleen meridian for 300 times, clearing the liver meridian for 250 times, rubbing Banmen for 120 times, rubbing Wailaogong for 100 times, and rubbing and pressing Zusanli for 120 times; chest pushing (including kneading, forked pushing, and straight-pushing Danzhong for 20 times each, as well as pressing between the ribs 5 times), rubbing Zhongwan and Dantian for 150 times each, back-pushing (including rubbing and pressing Feiyu for 150 times), pinching the spine 5 times, and pressing Jianjing 3 times. Children were massaged once a day, 4 times a week. All massages are done by the same pediatric massage therapist in our department. On this basis, the experimental group was given vitamin D drops (Qingdao Double Whale Pharmaceutical Co., Ltd., H20083372), 400 U/time, once a day. All children completed the 2-month treatment.

### Prognostic follow-up

All children were followed up for 6 months, using a regular review schedule that required each child to complete a review at least once a month. During the review, a study team member recorded whether the child experienced a recurrence of BA.

### Endpoints

(1) Symptom Relief Time: The time taken to relieve key symptoms, including wheezing, coughing, chest tightness, breathing difficulties, and wheezing rales, was documented.

(2) Immunological and Nutritional Biomarkers: Fasting venous blood samples were collected before and after treatment to assess the following markers: T lymphocyte subsets (CD3+, CD4+, CD8 + ) using flow cytometry. To evaluate the inflammatory response, interleukins (IL-4, IL-5, IL-10, and IL-13) were quantified by enzyme-linked immunosorbent assay (ELISA). Nutritional proteins, including albumin (ALB), transferrin (TRF), prealbumin (PA), and total protein (TP), were measured by a biochemical analyser to assess nutritional status.

Interleukins were analysed to examine the inflammatory pathway modulation by vitamin D supplementation and massage therapy concerning BA.

(3) Adverse Reactions: Any adverse reactions occurring during treatment were documented and analysed.

(4) Prognostic Recurrence Rate: Recurrence rate of BA in children during follow-up.

### Statistical methods

SPSS25.0 software was used for statistical analysis. The chi-square test was used for the comparison of counting data [n (%)], and independent sample t-test and paired t-test were used for the comparison of measurement data (x̄±s). The Kaplan-Meier method calculated the recurrence rate, and the survival rate was compared using the log-rank test. In addition, we validated the analytical results using the Bonferroni correction test. Results were considered statistically significant when P<0.05.

## Results

### There is no difference in the comparison of clinical data

Comparing the age, sex, disease type, and other clinical data, we found no statistical inter-group differences (P>0.05), confirming comparability (Table 1).

**Table 1:** Comparison of clinical data.

Groups (n=30)	Control	Experimental	χ^2^	P
Age	7.60±2.40	8.07±2.56	t=0.728	0.469
Boys	19 (63.33)	16 (53.33)	0.617	0.432
Girls	11 (36.67)	14 (46.67)		
Disease course of BA (months)	6.50±2.99	6.73±3.02	0.301	0.765
Allergic BA	17 (56.67)	20 (66.67)	0.666	0.717
Infectious BA	3 (10.00)	2 (6.67)		
Exercise BA	10 (33.33)	8 (26.67)		

### The experimental group shows a shorter time of symptom relief

**Figure 1:**
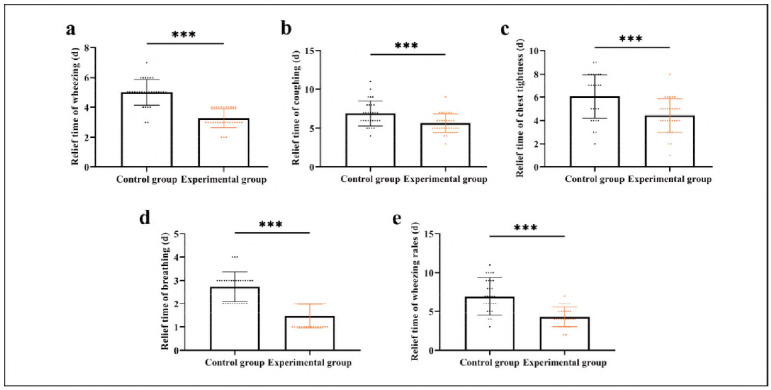
Comparison of time to symptom relief.

The statistical results are shown in Figure 1. The relief time of wheezing, coughing, chest tightness, breathing, and wheezing rales in the experimental group was shortened compared to the control group (P<0.001), indicating faster symptom improvement in the experimental group.

### The immune function and inflammatory response are better in the experimental group than in the control group

As shown in Table 2, the two groups were not markedly different in the results of T lymphocyte subsets before treatment (P>0.05). CD3^+^, CD4^+^, and CD4^+^/CD8^+^ in the experimental group all increased after treatment, higher compared to the control group, while CD8^+^ decreased and was lower (P<0.05), suggesting that the immune function of the experimental group was better. Additionally, IL-4, IL-5, IL-10, and IL-13 levels were measured before and after treatment. After treatment, the experimental group showed lower levels of IL-4 and IL-5 compared to the control group (P<0.05), indicating a reduced inflammatory response. However, no significant differences between the two groups were observed in IL-10 and IL-13 levels (P>0.05).

**Table 2:** Comparison of T-lymphocyte subsets.

Group (n=30)	Control	Experimental	t	P	
CD3+ (%)	Before treatment	58.52±3.98	59.37±4.94	0.732	0.467
	After treatment	64.93±5.32*	68.32±4.01*	2.788	0.007
CD4+ (%)	Before treatment	33.24±4.08	33.61±5.90	0.283	0.779
	After treatment	39.86±5.51*	44.99±8.57*	2.76	0.008
CD8+ (%)	Before treatment	34.20±3.26	33.76±4.08	0.465	0.644
	After treatment	29.65±3.72*	23.01±3.81*	6.831	<0.001
CD4+/CD8+	Before treatment	0.98±0.17	1.01±0.22	0.542	0.590
	After treatment	1.36±0.22*	2.01±0.51*	6.407	<0.001
IL-4 (pg/mL)	Before treatment	36.57±5.14	35.83±4.52	0.592	0.556
	After treatment	32.14±4.26*	28.29±3.11*	4.531	<0.001
IL-5 (pg/mL)	Before treatment	28.14±4.38	27.91±4.22	0.235	0.815
	After treatment	24.02±3.19*	22.03±2.57*	5.215	<0.001
IL-10 (pg/mL)	Before treatment	18.20±3.22	18.55±3.08*	0.430	0.669
	After treatment	24.68±3.56*	25.39±4.00*	1.130	0.263
IL-13 (pg/mL)	Before treatment	45.33±7.10	44.67±6.89	0.365	0.716
	After treatment	40.88±5.64*	39.52±4.77*	1.227	0.224

### The nutritional status of the experimental group is better than that of the control group

The results of nutritional protein are presented in Table 3. No notable inter-group differences were found in the detection results of nutritional proteins before treatment (P>0.05). After treatment, only ALB and TP in the control group increased, while all the tested nutritional proteins (ALB, TRF, PA, and TP) in the experimental group increased (P<0.05). The inter-group comparison revealed higher post-treatment ALB, PA, TRF, and TP in the experimental group (P<0.05), suggesting better nutritional status in the experimental group.

**Table 3:** Comparison of nutrient proteins. Note: * means P<0.05 compared to before treatment.

Groups (n=30)		Control	Experimental	t	P
ALB (g/L)	Before treatment	42.64±5.86	43.03±4.01	0.308	0.76
	After treatment	45.10±6.32*	48.36±5.19*	2.183	0.331
PA (mg/L)	Before treatment	345.09±41.57	340.94±26.07	0.463	0.645
	After treatment	343.55±23.28	363.00±38.13*	2.385	0.020
TRF (mg/L)	Before treatment	1.57±0.19	1.54±0.29	0.403	0.688
	After treatment	1.58±0.15	1.82±0.20*	5.357	<0.001
TP (g/L)	Before treatment	71.35±5.05	70.27±6.31	0.729	0.469
	After treatment	75.91±4.59*	78.33±3.63*	2.261	0.028

### There is no inter-group difference in safety

According to statistics, the incidence of adverse reactions in the experimental and control groups during treatment was 16.67% and 20.00%, respectively, with no significant difference (P>0.05), suggesting an equivalent safety profile (Table 4).

**Table 4:** Comparison of adverse reactions.

Groups (n=30)	Fever	Diarrhea	Nausea and vomiting	Headache	Total incidence
Control	2 (6.67)	1 (3.33)	2 (6.67)	1 (3.33)	20.00
Experimental	1 (3.33)	1 (3.33)	1 (3.33)	2 (6.67)	16.67
χ^2^					0.111
P					0.739

### The experimental group has a better prognosis than the control group

**Figure 2:**
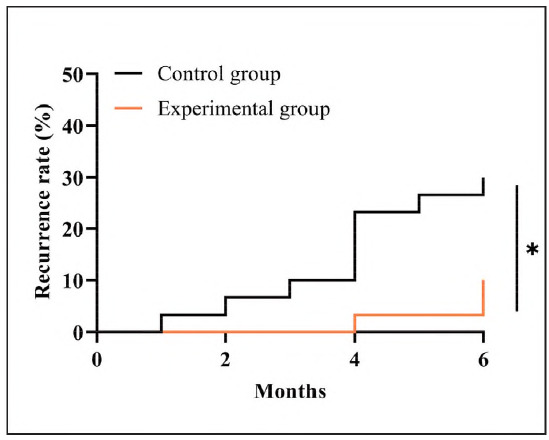
Comparison of prognostic recurrence.

Prognostic follow-up was successful in tracing all study participants. Statistics showed that the prognostic recurrence rate of the experimental group was 10.00%, which was notably reduced compared to the control group (P<0.05), indicating a better prognosis in the experimental group (Figure 2).

The prognostic BA recurrence rate was lower in the experimental group than in the control group. *P<0.05.

## Discussion

In this study, we found that pediatric massage combined with vitamin D effectively improved the nutritional status, immune function, and inflammatory response of children with BA and enhanced their prognosis and health, with extremely high clinical application value.

First of all, comparing the rehabilitation process of the two groups, it was found that the relief time of wheezing, coughing, chest tightness, breathing, and wheezing rales was shorter in the experimental group compared to the control group, indicating that the combination of pediatric massage and vitamin D can more quickly improve the clinical symptoms of BA children. In previous studies, using vitamin D improved the rehabilitation of COVID-19 patients [15], which is consistent with our results. As we all know, vitamin D is an essential nutrient for children's development, which can regulate cell growth and differentiation and hormone secretion, promote bone growth, and participate in multiple pathophysiological processes such as anti-infection and immune regulation [16]. Research has shown that vitamin D deficiency or insufficiency can contribute to airway disease occurrence and pathological progression by mediating airway remodelling and immune suppression [17]. Therefore, vitamin D supplementation can not only enhance the inhibitory effect of montelukast sodium on leukotriene activity but also further block the inflammatory cell infiltration pathway, alleviate the airway inflammatory reaction, and promote airway dilation, thus promoting airway health in BA patients [18]. Of course, the positive effect of pediatric massage cannot be ignored. Massage, as a traditional Chinese medicine treatment, stimulates the acupoints of the human body and adjusts the functions of viscera through manual stimulation, which has the functions of ventilating the lungs, depressing qi and relieving asthma, and resolving phlegm in the treatment of BA [19]. In previous studies, the effect of massage on improving children's cough has been demonstrated [20]. Therefore, we believe that pediatric massage as an adjunct to vitamin D can improve children's overall health, thereby enhancing their immune function, which is also the reason for optimising T lymphocyte subsets after treatment in the two groups.

However, in the comparison of nutritional status, only the experimental group showed improvement after treatment, while the control group showed no change, indicating that the combination of pediatric massage and vitamin D can improve the nutritional status of BA patients. Clinical research has confirmed that malnutrition is the main reason that affects the occurrence and progression of BA. This is because, in the chronic inflammatory state of BA, the body's immune system is overactivated, leading to increased energy consumption and imbalanced nutritional status, which further promotes immune dysfunction, exacerbates the development of BA, or leads to BA [21]. Vitamin D can promote the absorption of calcium and phosphorus, maintain calcium and phosphorus balance, and improve children's bone health [22]. Moreover, as a fat-soluble substance, vitamin D increases the permeability of intestinal mucosa to calcium ions, maintains the normal concentration of citrate in blood, and enhances the body's immunity [23]. Kokturk et al. [24] also found that vitamin D supplementation can regulate the functions of eosinophils, lymphocytes, mast cells, etc., inhibit excessive inflammatory responses, and promote the health of patients with chronic obstructive pulmonary disease. Under the above influence, the health status of the children in the experimental group was significantly improved, contributing to more significantly increased levels of nutritional proteins. Meanwhile, the absence of a significant difference in the incidence of adverse reactions between the two groups shows that vitamin D supplementation is highly safe and recommended for clinical use.

Additionally, the levels of key inflammatory cytokines, including IL-4, IL-5, IL-10, and IL-13, were measured before and after treatment to assess the impact of pediatric massage and vitamin D on immune regulation in children with BA. Our results showed that the experimental group had significantly lower levels of IL-4 and IL-5 after treatment, suggesting a reduction in the Th2-type inflammatory response, which plays a central role in the pathophysiology of BA. IL-4 and IL-5 are key cytokines that promote eosinophilic inflammation and mast cell activation, and elevated levels of both contribute to airway hyperresponsiveness, exacerbating the progression of BA [24]. The observed reduction in these cytokines indicates that the combined intervention of pediatric massage and vitamin D supplementation may help suppress the immune pathways that trigger these inflammatory cascades, improving clinical symptoms and respiratory function.

Furthermore, IL-13, another Th2 cytokine, was also reduced in the experimental group after treatment, although this reduction did not reach statistical significance. IL-13 is a key mediator in airway remodelling, mucus hypersecretion, and inflammation, and its modulation is crucial for long-term asthma control [7]. While the reduction in IL-13 in our study suggests some improvement in airway inflammation, this marker's lack of statistical significance may be due to individual variability in response or the relatively short treatment period. Interestingly, IL-10, a potent antiinflammatory cytokine, did not show significant changes between the groups. IL-10 is known for its immunosuppressive properties and ability to counteract the pro-inflammatory effects of cytokines like IL-4 and IL-5 [25] [26]. The lack of significant change in IL-10 levels in both groups may suggest that while the combined intervention successfully reduced the proinflammatory cytokines, it did not substantially affect the anti-inflammatory arm of the immune response. This finding could also indicate that the immunomodulatory effects of vitamin D and pediatric massage are more targeted at reducing the underlying inflammation associated with BA rather than enhancing IL-10-mediated immune tolerance. The results from the cytokine analysis support the hypothesis that the combination of pediatric massage and vitamin D supplementation can help modulate immune responses in children with BA. By reducing IL-4, IL-5, and IL-13 levels, the intervention may reduce eosinophilic inflammation, promote airway relaxation, and contribute to faster symptom relief and improved overall health. This immune modulation likely plays a significant role in the enhanced clinical outcomes observed in the experimental group, including reduced wheezing, improved lung function, and a lower recurrence rate of symptoms. These findings are consistent with previous studies suggesting that vitamin D supplementation has anti-inflammatory effects [27], which help control asthma exacerbations and improve longterm disease management. However, Guo M's study on the treatment of allergic rhinitis found that vitamin D use increased IL-10 in patients [28], which is different from the results of the current study. We analysed that this could be due to the different types of diseases (Guo M's study was on severe allergic rhinitis), in addition to the dose, frequency, and duration of vitamin D use, which could also affect this result. In the meta-analysis by El Abd A et al. [29], they also mentioned that vitamin D had no significant effect on eosinophils and IL-10 in the sputum of BA patients, which is the same as our results. We will conduct in vitro tests to verify the exact relationship between vitamin D and IL-10 as soon as possible.

Finally, in the follow-up investigation of prognosis, the experimental group also showed a lower recurrence rate of BA than the control group, indicating that the combination of pediatric massage and vitamin D is more helpful in maintaining the prognostic health of BA patients. This is also because vitamin D therapy can provide a more stable prognosis guarantee for children with BA, improve their health, and thus avoid the recurrence of BA. However, the number of cases included in this study is small, and the follow-up time is short, which does not rule out the possibility of statistical analysis contingency, so more comprehensive research is still needed for confirmation. Of course, as an allergic reaction disease, BA is affected by many factors (e.g., dust, pollen, animal hair, etc.) in the child's daily life. Since we cannot completely rule out the potential influence of these factors, we cannot be completely sure that vitamin D and pediatric massage reduce the prognostic recurrence rate of BA. In this regard, we need to conduct an in-hospital trial as soon as possible to confirm whether vitamin D and pediatric massage contribute to the reduction of prognostic recurrence of BA. Besides, we need to add more outcome measures to evaluate the overall impact of vitamin D on BA. In addition, unlike adults, treatment in children may be influenced mainly by treatment adherence. However, the current study did not investigate treatment adherence rates in children, which is an important limitation. Subsequently, we need to address these short-comings so that we can carry out more complete and in-depth research and analysis as soon as possible.

## Conclusion

The combination of pediatric massage and vitamin D can effectively improve children's immunity and nutritional status with BA and enhance their physical health, with extremely high clinical application value.

## Dodatak

### Availability of data and materials

The data used to support the findings of this study are available from the corresponding author upon request.

### Funding

No funds, grants, or other support was received.

### Acknowledgements

Not applicable.

### Conflict of interest statement

All the authors declare that they have no conflict of interest in this work.

## References

[1] Chetta A, Calzetta L (2022). Bronchial asthma: An update. Minerva Med.

[2] Qian K, Hongzhen X U, Chen Z, Zheng Y (2023). Advances in pulmonary rehabilitation for children with bronchial asthma. Zhejiang Da Xue Xue Bao Yi Xue Ban.

[3] Hammad H, Lambrecht B N (2021). The basic immunology of asthma. Cell.

[4] Thomas D, Mcdonald V M, Pavord I D, Gibson P G (2022). Asthma remission: what is it and how can it be achieved?. Eur Respir J.

[5] Chung K F, Dixey P, Abubakar-Waziri H, Bhavsar P, Patel P H, Guo S, Ji Y (2022). Characteristics, phenotypes, mechanisms and management of severe asthma. Chin Med J (Engl).

[6] Pelaia C, Heffler E, Crimi C, Maglio A, Vatrella A, Pelaia G, Canonica G W (2022). Interleukins 4 and 13 in Asthma: Key Pathophysiologic Cytokines and Druggable Molecular Targets. Front Pharmacol.

[7] Roeb E (2023). Interleukin-13 (IL-13)-A Pleiotropic Cytokine Involved in Wound Healing and Fibrosis. Int J Mol Sci.

[8] Qian G, Jiang W, Sun D, Sun Z, Chen A, Fang H, Wang J, Liu Y, Yin Z, Wei H, Fang H, Zhang X (2023). B-cell-derived IL-10 promotes allergic sensitization in asthma regulated by Bcl-3. Cell Mol Immunol.

[9] Matsuda M, Inaba M, Hamaguchi J, Tomita H, Omori M, Shimora H, Sakae H, Kitatani K, Nabe T (2022). Local IL-10 replacement therapy was effective for steroid-insensitive asthma in mice. Int Immunopharmacol.

[10] Carlberg C, Raczyk M, Zawrotna N (2023). Vitamin D: A master example of nutrigenomics. Redox Biol.

[11] Gaudet M, Plesa M, Mogas A, Jalaleddine N, Hamid Q, Al Heialy S (2022). Recent advances in vitamin D implications in chronic respiratory diseases. Respir Res.

[12] Ganmaa D, Enkhmaa D, Nasantogtokh E, Sukhbaatar S, Tumur O K E, Manson J E (2022). Vitamin D, respiratory infections, and chronic disease: Review of meta-analyses and randomized clinical trials. J Intern Med.

[13] Wang F, Jin L (2024). Research on the Mechanism and Application of Acupuncture Therapy for Asthma: A Review. J Asthma Allergy.

[14] Gupta N, Malhotra N, Kunal S, Ish P (2022). Management of bronchial asthma in 2021. Monaldi Arch Chest Dis.

[15] Chetty V V, Chetty M (2021). Potential benefit of vitamin D supplementation in people with respiratory illnesses, during the COVID-19 pandemic. Clin Transl Sci.

[16] Mathyssen C, Gayan-Ramirez G, Bouillon R, Janssens W (2017). Vitamin D supplementation in respiratory diseases: Evidence from RCT. Pol Arch Intern Med.

[17] Maes K, Serré J, Mathyssen C, Janssens W, Gayan-Ramirez G (2020). Targeting Vitamin D Deficiency to Limit Exacerbations in Respiratory Diseases: Utopia or Strategy With Potential?. Calcif Tissue Int.

[18] Battistini C, Ballan R, Herkenhoff M, Saad S, Sun J (2020). Vitamin D Modulates Intestinal Microbiota in Inflammatory Bowel Diseases.

[19] Wang C, Jiang Y, Fan Z, Zhao M, Jiang Y, Wang Z, Chen Z (2020). The efficacy of Tuina for asthma: A protocol for a systematic review and meta-analysis. Medicine (Baltimore).

[20] Mao H, Wei Y, Su H, Jiang Z, Li X (2022). Pediatric Tui Na for cough in children: A systematic review and meta-analysis of randomized controlled trials. Complement Ther Med.

[21] Alwarith J, Kahleova H, Crosby L, Brooks A, Brandon L, Levin S M, Barnard N D (2020). The role of nutrition in asthma prevention and treatment. Nutr Rev.

[22] Muszyński T, Polak K, Frątczak A, Miziołek B, Bergler-Czop B, Szczepanik A (2022). Vitamin D-The Nutritional Status of Post-Gastrectomy Gastric Cancer Patients-Systematic Review. Nutrients.

[23] Zhang J, Cao Z (2022). Exercise: A Possibly Effective Way to Improve Vitamin D Nutritional Status. Nutrients.

[24] Kokturk N, Baha A, Oh Y M, Young J J, Jones P W (2018). Vitamin D deficiency: What does it mean for chronic obstructive pulmonary disease (COPD)? A compherensive review for pulmonologists. Clin Respir J.

[25] Nagase H, Ueki S, Fujieda S (2020). The roles of IL-5 and anti-IL-5 treatment in eosinophilic diseases: Asthma, eosinophilic granulomatosis with polyangiitis, and eosinophilic chronic rhinosinusitis. Allergol Int.

[26] Saraiva M, Vieira P, O'garra A (2020). Biology and therapeutic potential of interleukin-10. J Exp Med.

[27] Lebiedziński F, Lisowska K A (2023). Impact of Vitamin D on Immunopathology of Hashimoto's Thyroiditis: From Theory to Practice. Nutrients.

[28] Guo M (2023). Vitamin D supplementation improves the therapeutic effect of mometasone on allergic rhinitis. Acta Biochim Pol.

[29] El Abd A, Dasari H, Dodin P, Trottier H, Ducharme F M (2024). The effects of vitamin D supplementation on inflammatory biomarkers in patients with asthma: A systematic review and meta-analysis of randomized controlled trials. Front Immunol.

